# A *TBK1* mutation disrupting IRF3 activation is associated with familial recurrent myopericarditis

**DOI:** 10.3389/fimmu.2026.1906965

**Published:** 2026-08-05

**Authors:** Claire J. Peet, Thomas Orchard, Carlos H. B. Cruz, Ariana Hernandez-Cordero, Ebun Omoyinmi, Satveer K. Mahil, Helen J. Lachmann, Franca Fraternali, Francesca Capon

**Affiliations:** 1 Department of Medical and Molecular Genetics, King’s College London, London, United Kingdom; 2 National Amyloidosis Centre, Royal Free London NHS Foundation Trust, London & Division of Medicine, University College London, London, United Kingdom; 3 Research Department of Structural and Molecular Biology, Division of Biosciences, University College London, London, United Kingdom; 4 St John’s Institute of Dermatology, King’s College London, London, United Kingdom; 5 Department of Biological Sciences, Birkbeck, University of London, London, United Kingdom; 6 BC Children’s Hospital Research Institute, Vancouver, BC, Canada; 7 Department of Medical Genetics and Centre for Molecular Medicine and Therapeutics, The University of British Columbia, Vancouver, BC, Canada

**Keywords:** IRF3, molecular dynamics, mutation, myopericarditis, TBK1, TLR3, recurrent pericarditis

## Abstract

**Introduction:**

Idiopathic recurrent pericarditis (IRP) is an immune-mediated disease characterized by episodes of pericardial inflammation. While IRP is increasingly considered an autoinflammatory condition, pathogenic pathways remain poorly understood. In this context, gene discovery can reveal new disease mechanisms.

**Methods:**

We carried out whole-exome sequencing in a pedigree where three individuals suffered from IRP presenting with myocardial involvement (myopericarditis). We first investigated mutation effects computationally, by estimating free-energy changes and undertaking molecular dynamic simulations of wild type and mutant proteins. Next, we overexpressed wild-type and mutagenized cDNAs in HEK293 cells, characterizing protein expression and phosphorylation by western blotting. Finally, we measured gene expression in primary pericardial fibroblasts, using real-time PCR.

**Results:**

We identified a Y105C mutation in TBK1, a kinase that regulates antiviral responses driven by the IRF3 transcription factor. The Y105C substitution maps at the interface between the kinase and scaffold domain of TBK1. Accordingly, molecular modeling predicted a destabilizing effect of the variant, which was experimentally validated in over-expression studies. The Y105C mutation also affected kinase activity, causing a significant reduction in IRF3 phosphorylation. While TBK1 was required for TLR3-IRF3 signaling in primary human pericardial fibroblasts, *TLR3* was weakly expressed in these cells.

**Conclusions:**

A TBK1 Y105C mutation that reduces IRF3 phosporylation is associated with familial recurrent myopericarditis. Given TLR3 is lowly expressed in pericardial fibroblasts, these cells may be especially vulnerable to mutations that disrupt TLR3-IRF3 signaling. Thus, our findings suggest that inherited susceptibility to viral infections may be an important factor in the pathogenesis of IRP.

## 1 Introduction

Idiopathic recurrent pericarditis (IRP) is a rare immune-mediated condition, characterized by acute episodes of pericardial inflammation (1). Disease flares present with severe chest pain, which can be accompanied by a pericardial friction rub, electrocardiographic changes, and/or pericardial effusion. In approximately 15% of cases, IRP is complicated by myocardial involvement, giving rise to myopericarditis (2).

While pericarditis recurrence has a profound effect on quality of life (3), the underlying risk factors and pathogenic pathways are not well understood. As IRP responds to drugs blocking inflammasome-dependent IL-1 activation (colchicine) or IL-1 signaling (anakinra, rilonacept) (4–6), an autoinflammatory pathogenesis has been proposed (7, 8). This is supported by several lines of evidence. First, pericarditis is a feature of autoinflammatory syndromes such as familial mediterranean fever and mevalonate kinase deficiency (9). Second, common *IL1B* variants have been associated with the disease in a genome-wide association study (10). Finally damaging changes in inflammasome genes (*NLRP3*, *MEFV*) have been detected among affected individuals (11).

Nonetheless, the mechanisms underlying abnormal IL-1 production in IRP are unclear. Studies of patient serum and pericardial effusions have suggested an involvement of viral infections, most notably those caused by enteroviruses (e.g. coxsackie B3 virus), influenza A virus and more recently, coronaviruses (12–15). In this context, disease flares might occur because of repeated infection, viral latency or priming of a subsequent autoinflammatory response. An alternative hypothesis is that non-infectious events (e.g. pericardial injury) might trigger the release of damage associated molecular patterns, leading to inflammasome activation, recruitment of innate immune cells and self-perpetuating inflammation (16, 17).

While various animal models of pericarditis have been established, they are of limited utility, as they cannot recapitulate the recurrent nature of IRP (18–20). Conversely, gene discovery in monogenic forms of the disease has unique potential to uncover pathogenic pathways.

Here, we investigated a recurrent myopericarditis kindred and uncovered a damaging mutation of the *TBK1* gene. We then showed that the disease allele disrupts the stability of the TBK1 protein and its ability to activate the IRF3 transcription factor. As IRF3 plays a critical role in innate antiviral responses, our findings implicate genetic susceptibility to viral infections in the pathogenesis of IRP.

## 2 Methods

### 2.1 Study participants

This study was conducted in accordance with the principles of the declaration of Helsinki. Ethical approval for the genetic analysis of IRP cases and their relatives was obtained from the Royal Free Hospital and University College Medical School Research Ethics Committee (ref 06/Q0501/42), while the use of discarded skin was approved by the London – Chelsea Ethics Committee (14/LO/2169). All participants granted their written informed consent.

### 2.2 Whole-exome sequencing

Whole-exome sequencing was undertaken on DNA samples obtained from three affected individuals and two unaffected family members. Paired-end reads were aligned to the hg19 reference genome using Novoalign (Novocraft Technologies) and Genome Analysis Toolkit (GATK; the Broad Institute). Variants calling was implemented with VCFtools and SAMtools (21). Sequence changes were then annotated using ANNOVAR (22).

After removing synonymous substitutions, variants were filtered to only keep those showing a minor allele frequency (MAF) <0.001 in the Exome Aggregation Consortium (ExAC) database (23). Changes that were not shared by all affected individuals were also removed. The pathogenicity of the remaining variants was assessed with multiple tools (CADD (24), SIFT (25), PolyPhen-2 (26) and MutationTaster (27) for coding changes; CADD and MutationTaster for splice site variants). Changes that were consistently classified as benign (at least 3 tools for coding variants, 2 for splice site changes) were removed. The genes harboring the variants that passed filtering were analyzed for genetic constraint (the deviation between observed and expected numbers of missense changes) by querying the z-scores reported in the gnomAD browser (v4.1.1) (28). Only genes yielding z-scores>3.0 were retained and assessed for biological relevance.

In a parallel analysis, Exomiser (29) was used to examine all variants that had a MAF<0.01 and were shared by the three affected individuals. The identification of the TBK1 Y105C variant was validated by Sanger sequencing using the primers reported in **Supplementary Table 1**. ACMG validator (https://switzerlandomics.ch/technologies/acmg-validator/) was used to confirm that the Y105C variant met the pathogenicity criteria defined by the American College of Medical Genetics and Genomics (30).

### 2.3 Computational analysis of *TBK1* variants

#### 2.3.1 Structure preparation and visualization

The experimentally resolved TBK1 structure (PDB 4IW0) was modified with PyMol (Schrödinger) as follows. The G1 and S2 residues (introduced for cloning purposes) were replaced with the original methionine. The catalytically inactivating D135N change (introduced to facilitate protein crystallization) was reversed, and the BX795 inhibitor was removed from the ATP binding site. The resulting structure was visualized using ChimeraX (31).

#### 2.3.2 Variant impact assessment

The pathogenicity of *TBK1* variants was examined using AlphaMissense (32), CADD and Rhapsody (33). Changes in folding free energy were assessed with FoldX (34), mCSM (35) and Rosetta (36). The latter analysis was implemented using the REF2015 energy function (37) on the ROSIE web server (38). Changes in standard ΔΔG were estimated with ThermoMPNN (39), while mCSM-PPI2 (40) was used to predict the change in binding free energy (ΔΔG) between the TBK1 kinase domain and scaffold/dimerization domain.

### 2.4 Molecular dynamics simulation

The TBK1 structure, with modifications described in 2.3.1, was first mutagenized in PyMOL to produce G159A and Y105C variant structures. The atom types were subsequently assigned to the ff14SB force field (41) using the AmberTools version 24.8 (42), with phosphorylated S172 defined by the phosaa14SB parameter set (43). Each variant TBK1 structure was embedded into a simulation box (10Å buffer) with TIP3P waters and nine Na+ ions to ensure neutrality. After energy minimization, the systems were heated to 300K through a temperature ramp of 20K every 40 ps in an NVT ensemble, with protein atom positions constrained by a harmonic potential with 5 kcal/mol·Å^2^ force constant. Unrestricted simulations were performed in an NPT ensemble at a constant pressure and temperature of 1 bar and 300K with 2 fs timestep over 1000 ns run-time using a Monte Carlo barostat and Langevin thermostat with 1 ps^-1^friction coefficient. In all simulations, a non-bonded cutoff of 1.0 nm was applied using a Particle Mesh Ewald framework. Five repeat simulations were performed for each variant condition to enhance conformational sampling and avoid dynamic artefacts.

The interface between the KD and SDD domains was analyzed by tracking interdomain distance and inter-domain residue contacts. For the interdomain distance analysis, the distance was calculated between the Cα of the two closest contacts at the interface between KD and SDD (Y/C105–Y424 and P101–G420) and subsequently converted into a Gaussian distribution using a Kernel Density Estimation function (44). For the contact analysis, pairwise contact was defined based on a 4.5 Å inter-residue cutoff distance, calculated as a frequency over 5 pooled repeats, and displayed as a contact heat map. All analyses were performed over the equilibrated trajectory using AmberTools and MDAnalysis (45).

### 2.5 Analysis of mutagenized *TBK1* constructs

#### 2.5.1 Construct generation

A pHAGE-N-FLAG-HA-TBK1 plasmid (Addgene) was used as a template for site directed mutagenesis. The reactions were carried out using the QuikChange Lightning Site Directed Mutagenesis Kit (Agilent) and the primers reported in **Supplementary Table 1**. The integrity of all constructs was verified by Sanger sequencing of the *TBK1* coding region, FLAG tag and CMV promoter.

#### 2.5.2 Culture and transfection of HEK293 cells

HEK293 cells were grown in in Dulbecco’s modified Eagle medium (DMEM) with GlutamaxTM (Gibco) supplemented with 10% fetal bovine serum (FBS) (Gibco) and 1% Penicillin/Streptomycin (Gibco). For overexpression experiments, cells were seeded at a density of 4x10^5^ cells/ml and transfected with 900ng plasmid DNA, using Lipofectamine 2000 Transfection Reagent (Thermo Fisher Scientific). Cells were harvested after 24 hours.

#### 2.5.3 Western blotting

Protein extracts were prepared by treating cells with non-denaturing lysis buffer (50mM TrisHCl pH 7.4, 150 mM NaCl, 10% glycerol, 5 mM EDTA) supplemented with 1x protease and phosphatase inhibitor (Cell Signaling Technologies). The proteins of interest were analyzed by western blotting, using the following primary antibodies: mouse polyclonal anti-FLAG (1:3000, Sigma); rabbit polyclonal anti-IRF3 (1:1000, Cell Signaling); rabbit polyclonal anti-pIRF3 (S386), (1:1000, Cell Signaling); rabbit monoclonal anti-CYLD (1:1000, Cell Signaling); rabbit polyclonal anti-pCYLD (S418) (1:1000, Cell Signaling); rabbit polyclonal anti-β actin (1:1000, Cell Signaling). Protein bands were quantified using ImageJ (46).

### 2.6 Analysis of primary fibroblasts

#### 2.6.1 Cell culture

Primary human pericardial fibroblasts were purchased from Caltag Medsystems (catalog #6430), while primary human dermal fibroblasts were isolated from healthy skin discarded after plastic surgery. Cells from four donors (two for each fibroblast type, all at passage 5) were grown in fibroblast medium supplemented with 2% fetal bovine serum, 1% fibroblast growth supplement, and 1% penicillin/streptomycin (all supplied by Caltag Medsystems).

For the poly(I:C) stimulation, pericardial fibroblasts were plated at a density of 1.5x10^5^ cells/ml, rested for 5h and then serum-starved for 10h. Cells were pretreated in triplicate with 10µM MRT67307 (Stratech Scientific) or vehicle. After 1h the medium was replaced with complete fibroblast medium supplemented with 10µg/ml high-molecular weight poly(I:C) (Invivogen) or vehicle. Cells were harvested 24h later.

#### 2.6.2 Gene expression analysis

Following RNA extraction and cDNA synthesis, real-time PCR was performed using the SYBR Green universal master mix (ThermoFisher Scientific) and the primers reported in **Supplementary Table 1**. Gene expression was normalized to *GAPDH* levels, using the ΔΔCT method.

### 2.7 Statistics

Gene expression values were log-transformed to correct for the log-normal distribution of RQ values produced by the ΔΔCT method. Differences between two groups were assessed using an unpaired t-test. Differences between three groups were analyzed using a one-way ANOVA, followed by Dunnett’s *post-hoc* test.

## 3 Results

### 3.1 Segregation of idiopathic recurrent myopericarditis in a nuclear family

**Figure 1:**
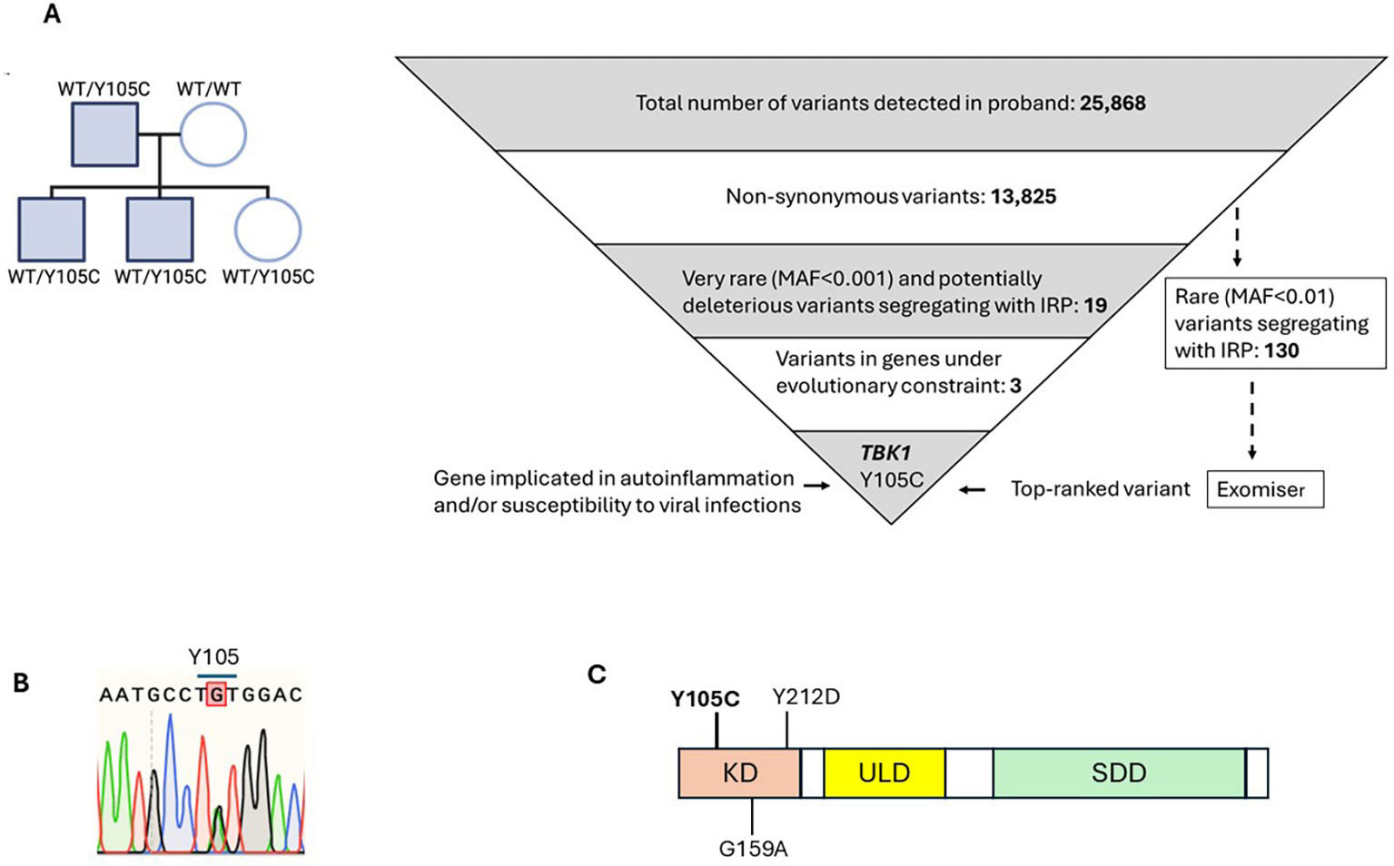
Identification of a c.A314G>Y105C *TBK1* variant in a myopericarditis pedigree. **(A)** Left: recurrent myopericarditis pedigree and segregation of the Y105C variant. Right: Workflow for the stepwise filtering of the whole exome sequence data. **(B)** Sanger sequencing chromatogram demonstrating heterozygosity for the c.A314G allele in the proband. **(C)** Schematic showing the location of the Y105C allele within the domain architecture of the TBK1 protein. The position of representative alleles associated with susceptibility to viral infection (G159A) and autoinflammation (Y212D) is also shown. KD, kinase domain; MAF, minor allele frequency; SDD, scaffold/dimerization domain; ULD, ubiquitin-like domain; WT, wildtype.

An analysis of the clinical records from National Amyloidosis Centre (Royal Free Hospital, London, UK) identified a family where three individuals suffered from recurrent myopericarditis (**Figure 1A**). The proband was a male of European descent, who presented with pericarditic chest pain at 17 and experienced a recurrence at 23. Based on characteristic electrocardiographic findings, raised inflammatory markers and elevated troponin levels (**Supplementary Table 2**), he received a diagnosis of IRP with myocardial involvement. The father and older brother of the proband also had a history of recurrent myopericarditis. The father was 21 when he experienced his first disease episode, which was followed by five confirmed recurrences. The brother first presented with myopericarditis at 16 and subsequently experienced three disease flares.

While the father reported a diarrheal illness prior to the onset of chest pain, there were no prodromal symptoms suggestive of viral infection in the other two cases. All myopericarditis episodes were managed with non-steroidal anti-inflammatory drugs (alone or with colchicine). Affected individuals were well between recurrences and had no other disease history. The proband’s mother and sister were both unaffected (**Figure 1A**).

### 3.2 Identification of a *TBK1* mutation by whole-exome sequencing

Whole-exome sequencing was undertaken in the three affected family members as well as the unaffected mother and sister. An average of 25,822 variants were detected in each study participant, with >90% of the target exome covered at a depth of 20X. Filtering was then implemented to only retain variants that were very rare (MAF<0.001), predicted to be deleterious by multiple classifiers (see Methods) and shared by the three affected individuals. This yielded 19 candidate mutations, which were further investigated by examining tolerance for missense changes in the relevant genes. Only three variants passed this filtering step, as they mapped to loci under significant genetic constraint: c.C3238CT>R1080C in *SOGA1*, c.C2312T>P767L in *DNM2* and c.A314G>Y105C in *TBK1*. While *DNM2* and *SOGA1* encode proteins that contribute to microtubule assembly (47, 48), *TBK1* is a mediator of antiviral immunity and inflammation (49). The Y105C *TBK1* variant was therefore considered the most likely disease-causing mutation (**Figure 1A**).

As the above result could have been influenced by the choice of filtering criteria and by our assumptions on disease pathogenesis, a parallel unbiased analysis was also undertaken. The allele frequency threshold was relaxed (MAF<0.01) and no pathogenicity predictors were used, yielding 130 variants shared by the three affected individuals. These were processed with Exomiser, a tool that annotates sequence variants and prioritizes them based on the phenotypic features of the disease of interest (29). Here, Exomiser identified the *TBK1* c.A314G>Y105C substitution as the top-ranked variant in the dataset. Conversely, neither the *SOGA1* nor the *DNM2* changes were among the top 10 variants.

The presence of the c.A314G>Y105C mutation was confirmed by Sanger sequencing of the three affected family members (**Figure 1B**). Of note, the change was also detected in the unaffected sister, suggesting reduced penetrance or delayed disease onset in this individual. Taken together, these findings identify the TBK1 Y105C substitution as a disease allele associated with familial recurrent myopericarditis.

### 3.3 Computational analysis of TBK1 variants

*TBK1* encodes a highly conserved serine threonine kinase which consists of an N-terminal kinase domain (KD), a ubiquitin-like domain and a C-terminal scaffold/dimerization domain (SDD) (**Figure 1C**). Interestingly, mutations mapping to the TBK1 KD have been associated with susceptibility to severe viral infection or autoinflammation (50–53). In this context, it is noteworthy that the Y105C myopericarditis mutation also maps to the protein KD (**Figure 1C**). We therefore decided to compare the effects of Y105C to those of representative variants associated with viral infection (G159A) and autoinflammation (Y212D). To this end, we performed a comprehensive computational analysis combining pathogenicity predictions, free energy evaluation, and molecular dynamics simulations.

**Figure 2:**
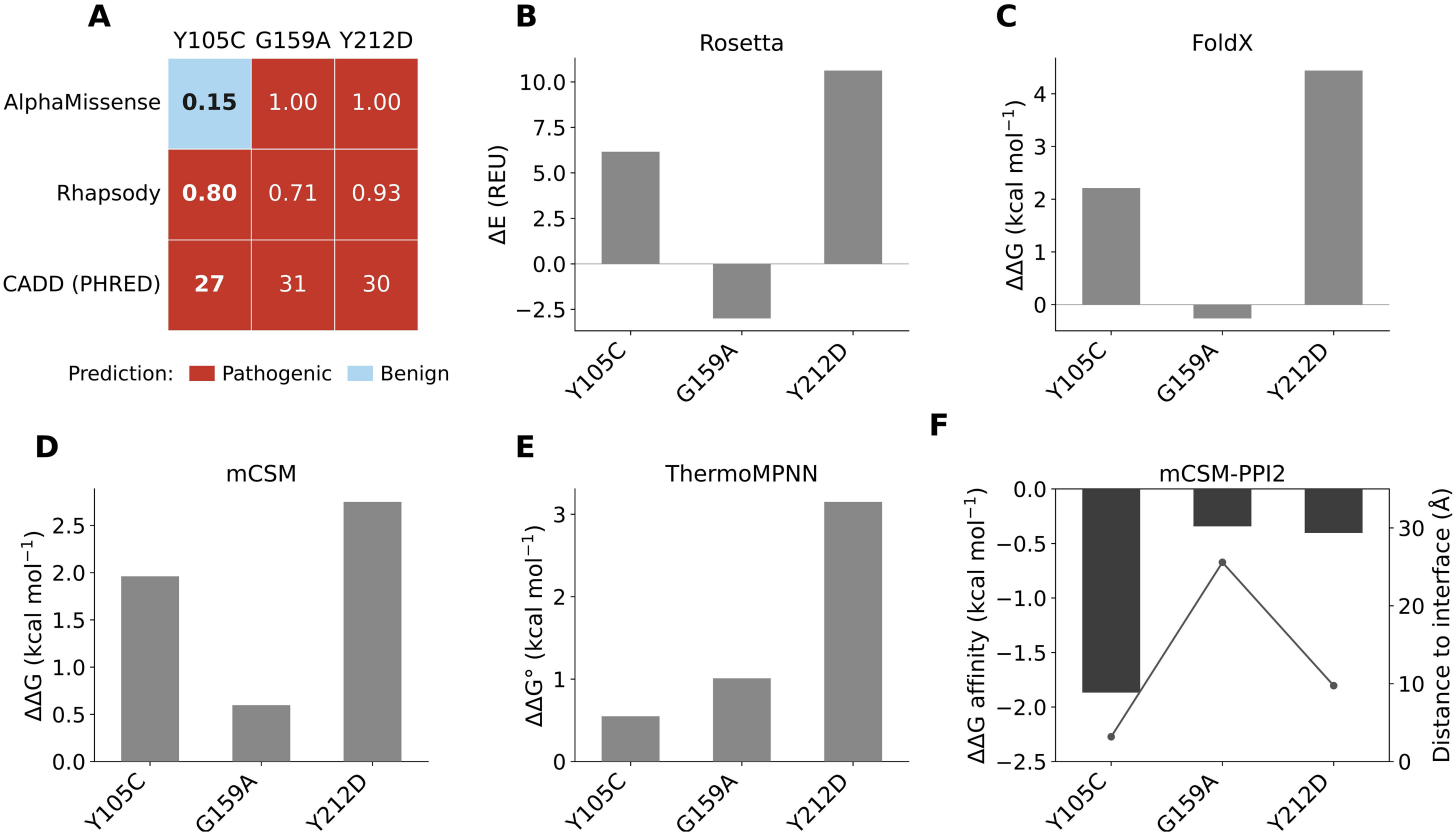
Predicted impact of the Y105C, G159A and Y212D TBK1 variants. **(A)** Application of machine learning variant classifiers to the three TBK1 variants. Cells are colored based on the predicted variant effect. Pathogenicity thresholds are reported in the Methods. **(B–E)** Predicted changes in energy resulting from the TBK1 variants. The bar plots show differences in Rosetta Energy Units (REU) **(B)**, folding free energy **(C, D)** and standard free energy of thermal unfolding **(E)**. Positive Δ values indicate decreased stability. **(F)** Bar plot showing the predicted change in binding free energy for the interaction between TBK1 KD and SDD. The distance between the mutation site and domain interface is illustrated as a line plot. Negative ΔΔG values indicate a reduction in inter-domain affinity.

Pathogenicity predictors (AlphaMissense, CADD, and Rhapsody) collectively supported a deleterious effect of the Y105C substitution, although with less consistency than for the G159A and Y212D variants (**Figure 2A**). In particular, the prediction of a likely benign effect by AlphaMissense suggests that the impact of Y105C may be more context-dependent and mechanistically subtle.

Consistent with this, multiple structure-based energy predictors (Rosetta, FoldX, mCSM, ThermoMPNN) indicated that Y105C moderately destabilized the TBK1 structure (**Figures 2B–E**). This was an intermediate effect: it exceeded the minimal impact observed for G159A but fell short of the strong destabilization predicted for Y212D (a variant that introduces a charged residue into the KD hydrophobic core).

**Figure 3:**
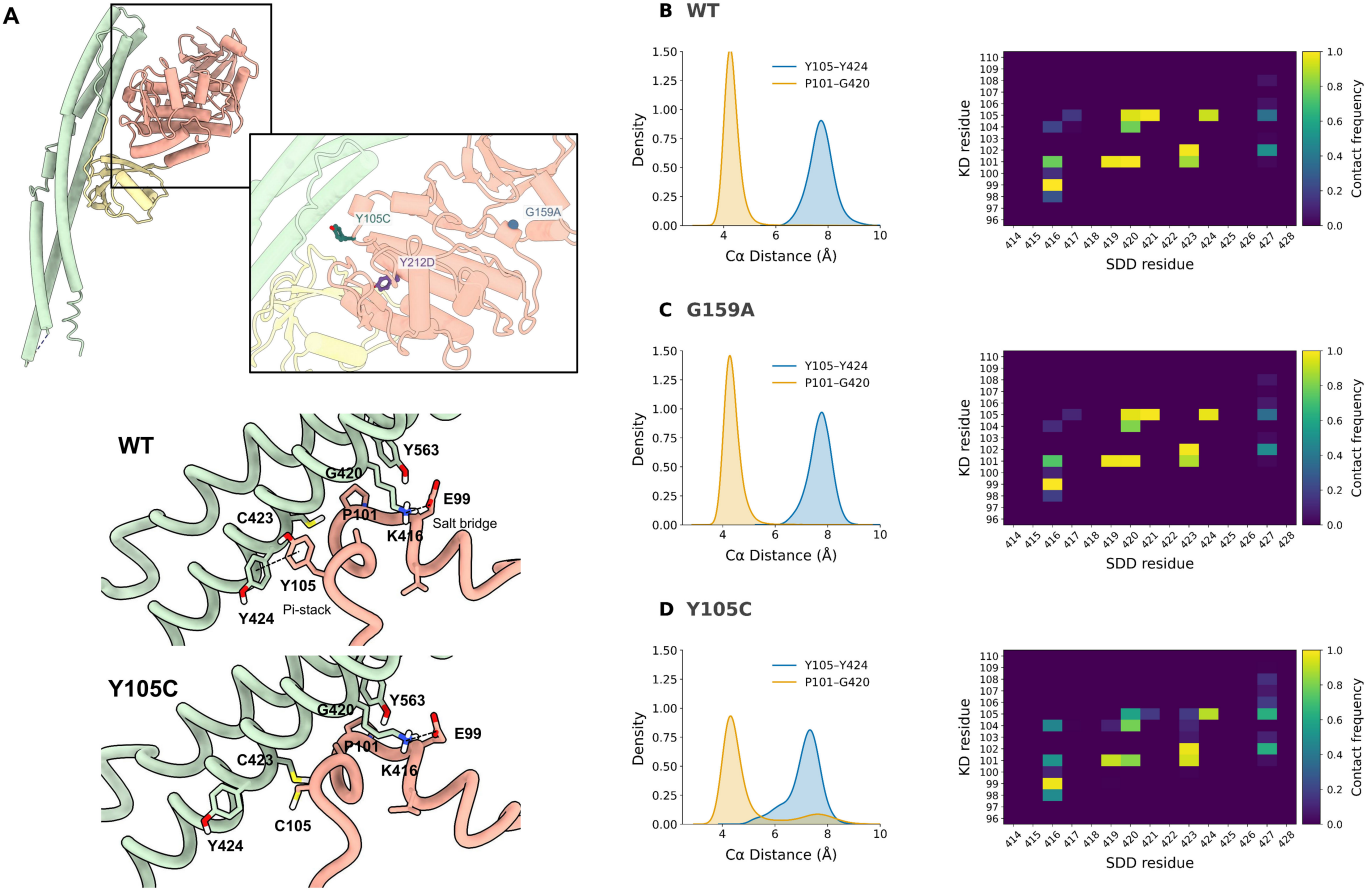
Contacts between the TBK1 kinase and scaffold/dimerization domains detected in molecular dynamic simulations. **(A)** Top: TBK1 protein structure with the scaffold/dimerization domain (SDD) colored in green, the kinase domain (KD) in orange and the ubiquitin-like domain in yellow. The expanded view of the kinase domain shows the location of the Y105C, G159A, and Y212D variants. Bottom: Enlarged view of the KD/SDD interface showing the effects of the Y105C mutation on interdomain interaction. **(B–D)** Results obtained in the simulations of wildtype **(B)**, G159A mutant **(C)** and Y105C mutant **(D)** TBK1. Left: distribution of the distance between the alpha carbon (Cα) atoms of selected amino acid residues at the closest points of contact between the KD and SDD. Each distribution is calculated over five 1000 ns simulations, with the first 100 ns omitted as equilibration. Right: contact frequency of residue pairs at the interface between the KD and SDD. Contact is defined as a distance < 4.5Å between the heavy atoms of the relevant residues. Contact frequencies were calculated across five simulation repeats.

Structural analysis further highlighted that the position of Y105 at the interface between the KD and SDD is critical for inter-domain stabilization. In the wild-type protein structure (PDB 4IW0), Y105 participates in a π-stacking interaction with Y424, which contributes to the integrity of the KD–SDD interface (**Figure 3A**). This interaction is abolished by the modeled Y105C substitution, and interaction energy calculations (mCSM-PPI2) predict a significant reduction in inter-domain affinity, a feature not observed for the other variants (**Figure 2F**).

These findings prompted us to perform molecular dynamics simulations to further investigate the impact of the Y105C substitution on the KD–SDD interface.

### 3.4 Molecular dynamics simulation

To further characterize the inter-domain interaction and compare the molecular features of wild-type and mutant structures, we performed molecular dynamics simulations of wild-type and mutant TBK1 models. These analyses revealed that the KD–SDD interface is maintained by three principal interaction regions, arranged along the axis connecting the kinase domain to the ubiquitin-like domain (**Figure 3A**).

The most prominent anchoring interaction, a salt bridge between E99 and K416, remained stable in the Y105C mutant protein, as indicated by unchanged hydrogen bond occupancy (**Figure 3A**) and contact frequency (**Figures 3B–D**). However, subtle rearrangements were detected in the surrounding interface, including increased hydrogen bonding between K416 and the adjacent residue L98, suggesting a local adjustment in domain geometry (**Figures 3B–D**).

More pronounced effects were observed at the central region of the interface, where contacts between P101 and G420 showed a reduction in frequency and a shift in the distance distribution. In the wild-type protein, this region is characterized by tight packing, whereas the Y105C mutant displayed a broader distribution with an additional peak at longer distances, consistent with a partial loosening of inter-domain interactions (**Figures 3B–D**). At the distal end of the interface, the Y105 residue normally participates in a π-stacking interaction with Y424. This interaction was abolished by the Y105C substitution, resulting in altered contact patterns and a subtle repositioning of the kinase domain relative to the scaffold domain. Despite this loss, interface residues rearranged their contacts and the two proteins remained in contact indicating that the mutation induces a geometric reorganization of the two proteins’ interface rather than complete disruption of domain association (**Figure 3A**).

Taken together, these findings indicate that the Y105C mutation does not alter the primary anchoring interaction between TBK1 domains but rather induces localized destabilization and reconfiguration of the KD–SDD interface. This structural perturbation suggests reduced protein stability and supports a model in which altered domain coupling contributes to impaired TBK1 function.

### 3.5 The Y105C mutation disrupts TBK1 stability and IRF3 phosphorylation

**Figure 4:**
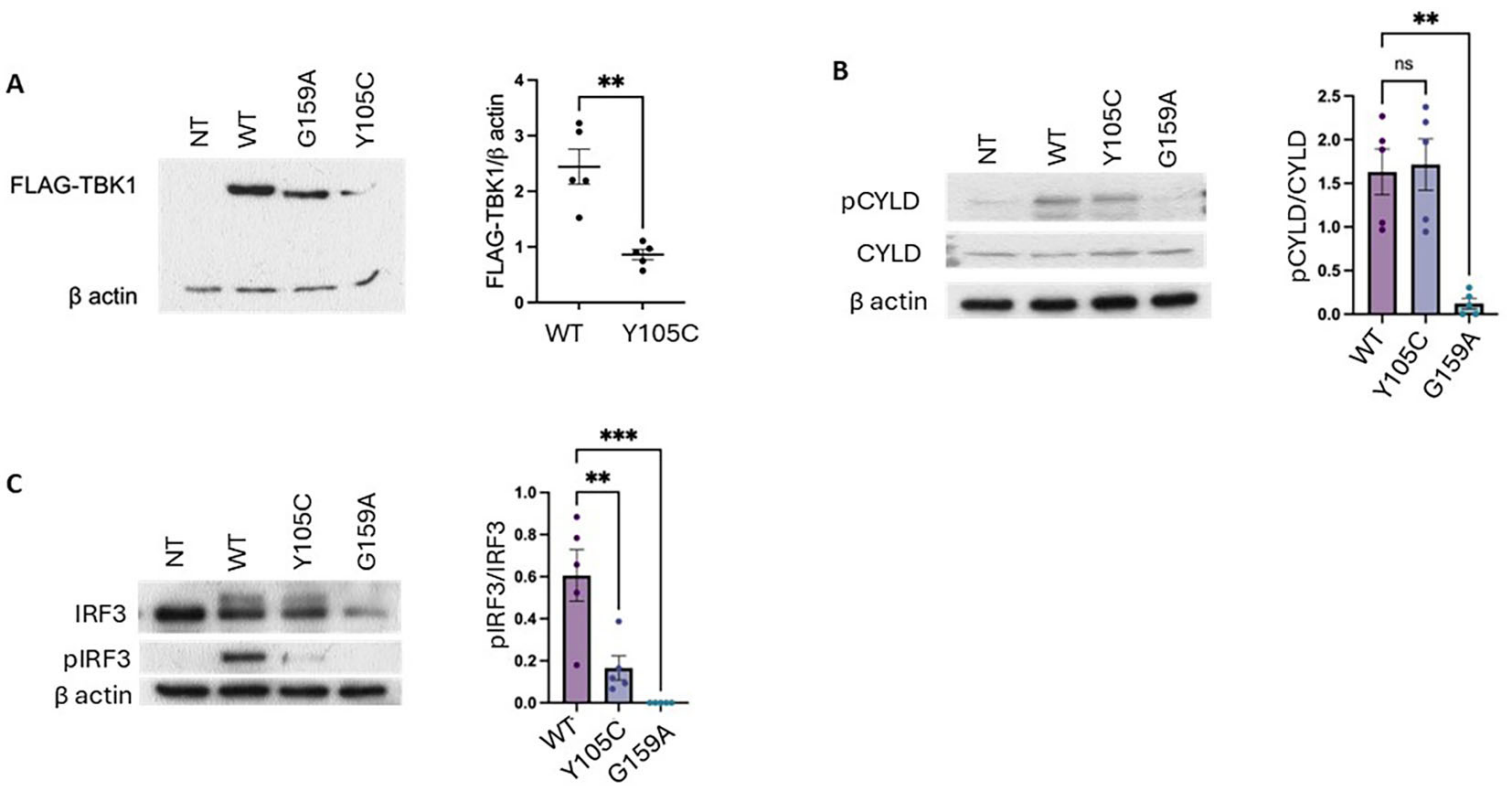
The Y105C variant affects TBK1 stability and function. **(A)** Left: representative western blot showing the accumulation of wildtype (WT) and mutant FLAG-TBK1 in HEK293 cells. Right: quantification of WT and Y105C bands. **P < 0.01 (Student’s t-test). **(B)** Left: representative western blot showing the accumulation of phosphorylated CYLD (pCYLD) in HEK293 cells transfected with wildtype (WT) or mutant FLAG-TBK1. Right: quantification of pCYLD/CYLD ratios normalized to β actin. **P < 0.01 (one-way ANOVA with Dunnet’s post-test). **(C)** Left: representative western blot showing the accumulation of phosphorylated IRF3 (pIRF3) in HEK293 cells transfected with wildtype (WT) or mutant FLAG-TBK1. Right: quantification of pIRF3/IRF3 ratios normalized to β actin. **P < 0.01, ***P < 0.001 (one-way ANOVA with Dunnet’s post-test). In all bar plots, data are shown as mean +/- SD of results obtained in five independent transfections. ns, not significant; NT, not transfected.

To experimentally validate the effect of the Y105C mutation on TBK1 stability, we transfected wild-type or mutant cDNA constructs in HEK293 cells. We then measured TBK1 protein levels by western blot. This showed that the expression of Y105C-TBK1 was detectable, but consistently reduced compared to wild-type, in keeping with a moderate destabilizing effect of the variant. Of note, the G159A change, which is not expected to disturb protein folding (50, 52), did not affect TBK1 protein expression (**Figure 4A**).

We next examined the possibility that the Y105C substitution might affect the protein’s kinase activity. We therefore measured the phosphorylation of selected TBK1 targets in HEK293 cells. Since TBK1 regulates antiviral responses as well as proinflammatory cell death (necroptosis) (49), we examined one target associated with each pathway.

We first investigated the phosphorylation of CYLD, an event that suppresses necroptosis and the ensuing release of molecules (damage associated molecular patterns) that can activate the inflammasome (52). As expected, we found that phosphorylated (p) CYLD was virtually undetectable in cells transfected with G159A-TBK1 (**Figure 4B**), which has been characterized as a catalytically inactive mutant (50). Conversely, the Y105C mutation had no effect on the accumulation of pCYLD (**Figure 4B**). Next, we examined the phosphorylation of IRF3, a transcription factor that mediates type I interferon (IFN) production in response to viral nucleic acids. We observed that both the Y105C and G159A mutation caused a reduction in pIRF3 levels, although the latter had a more profound effect (**Figure 4C**).

These observations show that the TBK1 mutation associated with recurrent myopericarditis (Y105C) destabilizes protein structure and selectively disrupts IRF3 phosphorylation. Of note, these functional readouts confirm that the variant meets the pathogenicity criteria established by the American College of Medical Genetics and Genomics (30) (**Supplementary Table 3**).

### 3.6 Innate antiviral receptors are weakly expressed in human pericardial fibroblasts

Given the results of our *in-vitro* experiments, we sought to further investigate the effects of TBK1 impairment on IRF3 signaling. TBK1 acts downstream of pattern recognition receptors that sense viral nucleic acids. It phosphorylates IRF3, driving the expression of type I IFN and other antiviral molecules (49). While TBK1 can be activated by multiple receptors, we reasoned that Toll-like receptor 3 would be the most relevant to the pathogenesis of myopericarditis, as it mediates immune responses against the coxsackie virus (54).

**Figure 5:**
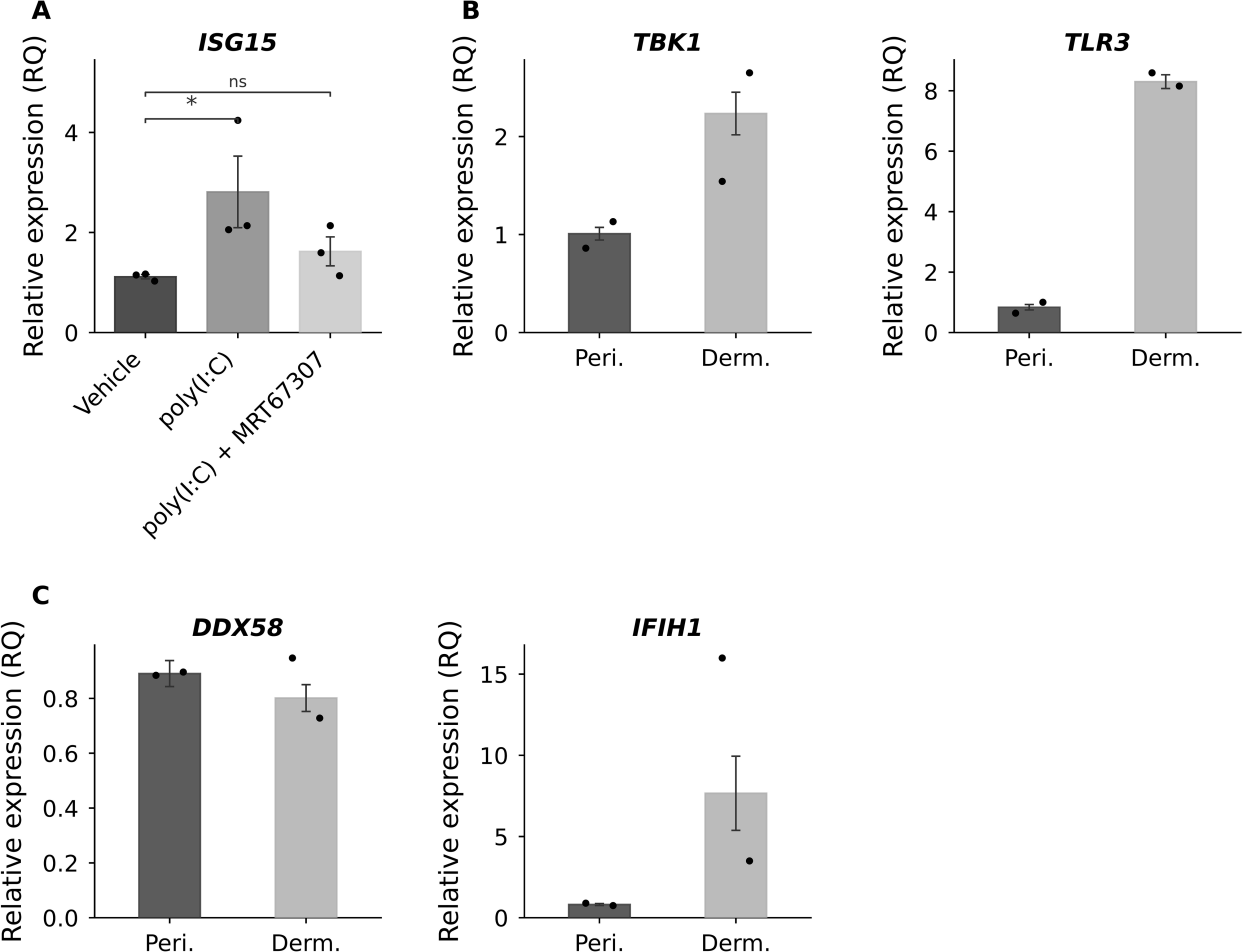
*TBK1* signaling activity and antiviral receptor expression in primary fibroblasts. **(A)** Real-time PCR analysis of primary pericardial fibroblasts showing the effects of poly(I:C) treatment in the presence or absence of the MRT67307 TBK1 inhibitor. Gene expression is normalized to *GAPDH* levels and data are presented as the mean +/- SD of three technical replicates. *P < 0.05 (one-tailed Student’s t-test); ns, not significant. **(B, C)** Real-time PCR analysis of *TBK1*, *TLR3* **(B)** *IFIH1* and *DDX58* **(C)** expression in pericardial (Peri) vs dermal (Derm) fibroblasts. Gene expression levels are normalized as above. Data are presented as the mean +/- SD of results obtained in two independent donors per cell type.

To interrogate TLR3/IRF3 signaling in an appropriate context, we stimulated human primary pericardial fibroblasts. We treated these cells with poly(I:C) (a synthetic TLR3 ligand) and then measured the expression of *ISG15*, a key IRF3 target gene. As expected, we observed that poly(I:C) treatment upregulated *ISG15* mRNA levels. This increase, however, could be eliminated by pre-treating the cultures with a pharmacological TBK1 inhibitor (MRT67307) (**Figure 5A**). Thus, impaired TBK1 function disrupts TLR3/IRF3 signaling in pericardial cells.

We noticed that poly(I:C) stimulation upregulated *ISG15* levels by less than three-fold (**Figure 5A**). To investigate the reasons underlying this weak response, we measured *TBK1* and *TLR3* mRNA expression in pericardial fibroblasts. We used dermal fibroblasts as a comparator, as innate antiviral responses are well characterized in these cells (55). This analysis showed that in pericardial fibroblasts, *TBK1* levels are reduced by 2-fold and *TLR3* levels by 8-fold, compared to dermal fibroblasts (**Figure 5B**). To assess whether other innate antiviral receptors were similarly downregulated, we measured the expression of *DDX48*/RIG-I and *IFIH1*/MDA5, as both can activate TBK1 in response to viral nucleic acids. This showed that *IFIH1* mRNA levels are also reduced in pericardial vs dermal fibroblasts (**Figure 5C**). Thus, innate antiviral receptors acting upstream of TBK1 are lowly expressed in pericardial fibroblasts, suggesting that these cells might be especially vulnerable to the effects of TBK1 disruption.

## 4 Discussion

The aim of this study was to uncover new determinants of IRP through the genetic analysis of a multiplex pedigree. In this context, stepwise filtering of whole-exome sequence data identified a *TBK1* mutation as the most likely disease allele. While this result was independently validated by the Exomiser program, we cannot exclude the possibility that changes in additional genes may have contributed to the disease phenotype or accounted for the reduced penetrance of the Y105C mutation. For example, the three affected individuals -but not the unaffected sister - share a P1089L substitution in *THBS2*. This gene has a protective role in murine myocarditis, as *Thbs2* -/- mice upregulated cardiac inflammation following coxsackie virus infection (56). Thus, it is tempting to speculate that the heterozygous P1089L change might modify the effects of the *TBK1* mutation in humans.

Meanwhile, the pathogenic role of TBK1 is supported by its dual function as regulator of antiviral responses and inhibitor of inflammasome activation (49). It is also underscored by the effects of known *TBK1* mutations. These can cause increased susceptibility to viruses such as SARS-Cov2 and HSV-1 (50, 51, 53), which have been linked to pericarditis and myopericarditis (14, 57).

*TBK1* mutations have also been associated with neuroinflammation, as they have been repeatedly detected in cases of amyotrophic lateral sclerosis and frontotemporal dementia (58). Thus, *TBK1* alleles cause a multitude of immune-mediated phenotypes, which suggests that they may have varying impacts on protein structure and function. In this context, our initial study of free-energy changes confirmed substantial differences in the effects of the Y105C (moderately destabilizing), G159A (not destabilizing) and Y212D (very destabilizing) mutations. Likewise, our analysis of the experimentally resolved TBK1 structure localized the Y105C variant (but not G159A or Y212D) to the interface between the kinase and scaffold domains. Molecular dynamics simulations indicated that the Y105C mutation specifically destabilizes the interaction between these two domains, with potential to disrupt the positioning and phosphorylation of TBK1 targets. This suggests that Y105C has a nuanced effect on kinase function, disrupting conformational coupling and complex formation rather than abolishing catalytic activity.

These computational predictions are borne out by the results of our *in-vitro* experiments. We observed that the Y105C-mutant protein is markedly less stable than its wild-type counterpart. We also showed that the mutation impairs the phosphorylation of IRF3 while preserving that of CYLD. This is in contrast with the effects of G159A, which completely abolishes kinase activity (50). While we did not examine additional TBK1 targets, others have shown that mutations affecting the kinase domain have varying effects on the phosphorylation of p62 and OPTN (59).

Differences in functional impact, however, cannot fully account for the diversity of the phenotypes associated with *TBK1* alleles. For example, the Y105C substitution has also been observed in one patient with amyotrophic lateral sclerosis (60). *In-vitro* follow-up of the variant showed reduced activation of IRF3 signaling (59), in keeping with our observation of decreased IRF3 phosphorylation. While clinical information was limited, there was no indication that the affected individual had ever experienced pericarditis.

In this context, the difference in clinical outcomes could reflect the role of modifier genes (e.g. *THBS2*, as discussed above) or differences in environmental triggers. Inter-individual variability in the expression of TBK1 and its signaling partners could also be a factor. In fact, we observed that *TBK1* and *IFIH1* mRNA levels varied across fibroblast donors.

Finally, it is worth bearing in mind that genotype-phenotype correlations evolve as sample sizes grow and clinical follow-up becomes more detailed. When the *TBK1* mutations associated with severe autoinflammation were first identified, susceptibility to viral infection was not considered part of the phenotypic spectrum (52). A recent update, however, reported various instances of severe or lethal infection, which were uncovered as new cases were identified and known patients were continuously monitored (61, 62).

Our study has some limitations. The *TBK1* Y105C variant was observed in a single pedigree, so disease causation cannot be conclusively demonstrated. This is reflected by the application of American College of Medical Genetics and Genomics criteria, which classify the variant as “likely pathogenic” (**Supplementary Table 3**). Likewise, we could not provide direct evidence of viral involvement in the affected individuals, owing to the historical nature of disease flares. Thus, the link between impaired antiviral responses and IRP remains unproven, albeit highly plausible.

In this context, the genetic analysis and clinical characterization of additional IRP pedigrees will be required to further investigate the pathogenic role of TBK1 alleles. Such studies hold the promise to improve our understanding of pericarditis recurrence.

## Data Availability

The original contributions presented in the study are included in the article/**Supplementary Material**. Further inquiries can be directed to the corresponding authors.
