# Supplementary material for: A TBK1 mutation disrupting IRF3 activation is associated with familial recurrent myopericarditis
Source: Front Immunol. 2026 Aug 5;17:1906965. doi: 10.3389/fimmu.2026.1906965 (PMC13485522; doi:10.3389/fimmu.2026.1906965)
Supplement: Supplementary file 1 [file Table1.docx]

Supplementary Material

# 1 Supplementary Tables

**Supplementary Table 1:** Primer details

| *Application* | *Sequence (5’- 3’)* |
| --- | --- |
| Validation of *TBK1* Y105C mutation | F: TGAATCTCAGGTGAAGCAAAGT  R: GCATTGCATAAAGGTCTTCAGTT |
| *TBK1* mutagenesis (Y105C) | F: GAAGAACCTTCTAATGCCTGTGGACTACCAGAATCTG  R: CAGATTCTGGTAGTCCACAGGCATTAGAAGGTTCTTC |
| *TBK1* mutagenesis (G159A) | F: CTGTGTACAAACTCACAGATTTTGCTGCAGCTAGAGAATTAGAAG  R: CTTCTAATTCTCTAGCTGCAGCAAAATCTGTGAGTTTGTACACAG |
| *DDX58* real-time PCR | F: TATGTGCTCCTACAGGTTGTGG  R: GACAACTTTCCCCTTTTGTCCT |
| *GAPDH* real-time PCR | F: CGGAGTCAACGGATTTGGTC  R: AATGAAGGGGTCATTGATGGCA |
| *IFIH1* real-time PCR | F: CAGATGCAACCAGAGAAGATCCA  R: GCCCATTGTTCATAGGGTTGAGT |
| *ISG15* real-time PCR | F: GAGCACCGTGTTCATGAATCTG  R: TTATTTCCGGCCCTTGATCCTG |
| *TBK1* real-time PCR | F: GCTAGCATGGCTAAGGCAATAAC  R: CCATCGTATCCCCTTTCGCATTA |
| *TLR3* real-time PCR | F: GCGCTAAAAAGTGAAGAACTGG  R: TTCCAATTGCGTGAAAACACCC |

**Supplementary Table 2**: Presentation of recurrent myopericarditis in the proband

|  | **First episode** | **Second episode** |
| --- | --- | --- |
| *Age* | 17 | 23 |
| *Symptoms* | Severe central chest pain, relieved by sitting forward | Severe central chest pain, relieved by sitting forward |
| *Management* | Ibuprofen | Ibuprofen, colchicine |
| *White cell counts* | 7.6x10^9^/L | 11.8 x10^9^/L |
| *Neutrophil counts* | 6.3x10^9^/L | 8.4 x10^9^/L |
| *Neutrophil to leukocyte ratio* | 6.2 | 5.2 |
| *C-reactive protein* | 11 mg/L | 7 mg/L |
| *Troponin T* | 760 ng/L | 481 ng/L |
| *C3, C4* | Normal | Normal |
| *Electrocardiogram* | Global ST elevation & PR depression | Global ST elevation & PR depression |
| *Transthoracic echocardiogram* | Normal (at 2 months) | Normal (at 3 months) |

**Supplementary Table 3a:** Evidence review for the Y105C *TBK1* variant

| **Pathogenic evidence** |  |  |  |
| --- | --- | --- | --- |
| *ACMG Criterion^1^* | *Status* | *Applied strength* | *Points* |
| **PS3**: Well-established functional studies show a damaging effect. | Present | Moderate^2^ | +2 |
| **PM2**: Absent, or at extremely low frequency, in population databases | Present | Moderate | +2 |
| **PP1**: Co-segregation with disease in multiple affected family members. | Present | Supporting | +1 |
| **PP2**: Missense in a gene with a low rate of benign missense. | Present | Supporting | +1 |
| **PP3**: Computational evidence supports a deleterious effect | Present | Supporting | +1 |
|  |  |  |  |
| **Benign evidence** |  |  |  |
| *ACMG Criterion^1^* | *Status* | *Applied strength* | *Points* |
| **BA1**: Allele frequency above the disease-appropriate stand-alone benign threshold | Absent | Stand-alone | -- |
| **BS1**: Allele frequency greater than expected for the disorder | Absent | Strong | -- |
| **BS2**: Observed in a healthy adult where full penetrance is expected at an early age | Absent | Strong | -- |
| **BS3**: Well-established functional studies show no damaging effect | Absent | Strong | -- |
| **BS4**: Lack of segregation in affected family members | Absent | Strong | -- |
| **BP1**: Missense in a gene where only truncating variants cause disease | Absent | Supporting | -- |
| **BP2**: Observed in trans with a pathogenic variant in a fully penetrant dominant gene | Absent | Supporting | -- |
| **BP4**: Computational evidence supports no impact | Absent | Supporting | -- |
| **BP5**: Variant found in a case with an alternate molecular basis for disease. | Absent | Supporting | -- |
| **BP6**: Reputable source reports benign but evidence is unavailable. | Absent | Supporting | -- |

ACMG, American College of Medical Genetics and Genomics; ^1^Criteria that were not applicable to the Y105C variant were excluded from the review;^2^The number of points was reduced from 4 to 2, as CYLD phosphorylation was preserved

**Supplementary Table 3b:** Outcome of evidence review for the Y105C *TBK1* variant

| *Pathogenic points* | 7 |
| --- | --- |
| *Benign points* | 0 |
| *Total* | 7 |
| *Point-based category* | **Likely Pathogenic** |
